# Archaea: A Gold Mine for Topoisomerase Diversity

**DOI:** 10.3389/fmicb.2021.661411

**Published:** 2021-05-25

**Authors:** Florence Garnier, Mohea Couturier, Hélène Débat, Marc Nadal

**Affiliations:** ^1^Département de biologie, Institut de Biologie de l’Ecole Normale Supérieure (IBENS), École normale supérieure, CNRS, INSERM, Université PSL, Paris, France; ^2^Université Paris-Saclay, UVSQ, Versailles, France; ^3^Carl R. Woese Institute for Genomic Biology, University of Illinois at Urbana-Champaign, Urbana, IL, United States; ^4^Université de Paris, Paris, France

**Keywords:** topoisomerase, topology, archaea, extremophiles, genome stability, replication, repair, recombination

## Abstract

The control of DNA topology is a prerequisite for all the DNA transactions such as DNA replication, repair, recombination, and transcription. This global control is carried out by essential enzymes, named DNA-topoisomerases, that are mandatory for the genome stability. Since many decades, the Archaea provide a significant panel of new types of topoisomerases such as the reverse gyrase, the type IIB or the type IC. These more or less recent discoveries largely contributed to change the understanding of the role of the DNA topoisomerases in all the living world. Despite their very different life styles, Archaea share a quasi-homogeneous set of DNA-topoisomerases, except thermophilic organisms that possess at least one reverse gyrase that is considered a marker of the thermophily. Here, we discuss the effect of the life style of Archaea on DNA structure and topology and then we review the content of these essential enzymes within all the archaeal diversity based on complete sequenced genomes available. Finally, we discuss their roles, in particular in the processes involved in both the archaeal adaptation and the preservation of the genome stability.

## Introduction

**FIGURE 1:**
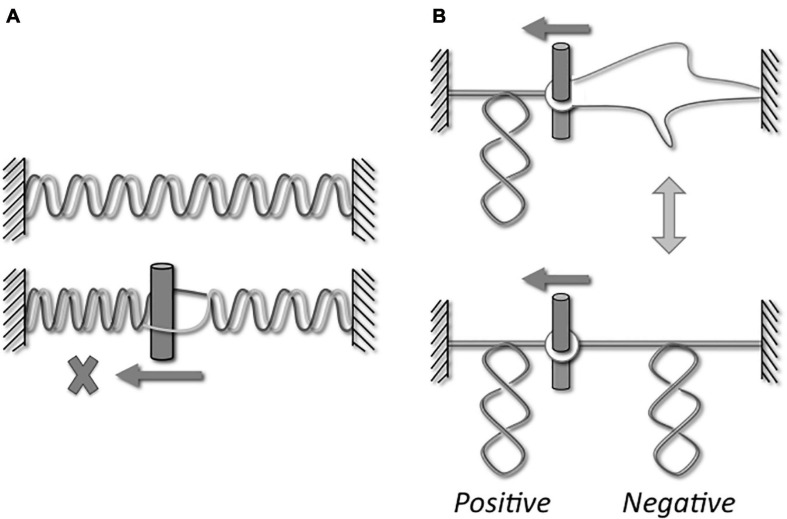
Topological stresses during dynamic processes. If the DNA machineries do not rotate, their movements along the DNA induce an overwinding of the DNA ahead the machineries (**A**, lower part). Considering the rigidity of the DNA, the torsional stresses cannot significantly modify the helical repeat and, consequently, it is the axis that forms supercoils. Indeed, a positive supercoiling appears ahead the machineries (**B**, upper and lower parts). A melted DNA region can be formed behind the machineries (**A**, lower part). However, if the temperature is lower than the DNA melting temperature, the DNA strands are reannealed to form a double helical structure. As a consequence of this underwinding, the DNA axis forms negative supercoils (**B**, lower part). The equilibrium that exists between a melted form and negative supercoils form depends on the temperature and the DNA sequence. The consequence of this over- and underwinding is the arrest of the machineries or the loss of DNA recognition, respectively.

**FIGURE 2:**
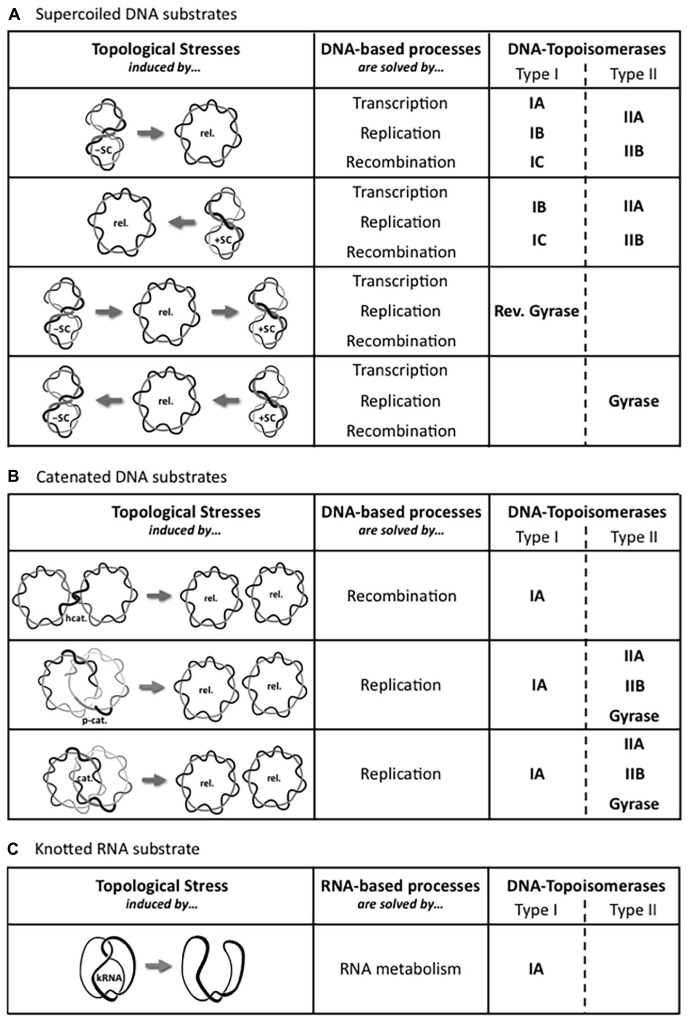
Set of DNA-topoisomerases involved depending on the topological stress. The movement of DNA-based processes, such as transcription, DNA replication, and recombination, locally induces different topological stresses, which are schematically represented. These local torsional stresses have been observed within **(A)** Supercoiled DNA substrates formed within a single molecule of double-stranded DNA, between **(B)** Catenated DNA substrates involving two molecules of double-stranded DNA, or within **(C)** Knotted RNA substrate formed within a single molecule of single-stranded RNA, and are solved by either type I or type II DNA-topoisomerases, or by both types. The two types are subdivided into their respective subfamilies: IA, IB, and IC; IIA and IIB. The gray arrow represents the DNA-topoisomerase activity. Abbreviations designing topological forms are: relaxed DNA (Rel.), negatively supercoiled DNA (-SC), positively supercoiled DNA (+SC), pre-catenane (p-cat.), catenane (cat.), hemicatenane (hcat.), and knotted RNA (kRNA).

All living organisms use DNA as the carrier of the genetic information but, as noticed by Watson and Crick (Watson and Crick, 1953), the double helical structure of the B-DNA intrinsically raises a big issue for its dynamics. Indeed, as early as they proposed this structure for DNA, they wrote: “Since the two chains in our model are intertwined, it is essential for them to untwist if they are to separate.” In addition, they wondered the following point: “What makes the pair of chains unwind and separate?” Thus, many different topological problems are triggered during DNA metabolism as illustrated in the Figure 1. Indeed, strand separation is required in all the DNA processes, such as genome replication, gene transcription, DNA repair and recombination. Strands separation induces a torsional stress in the DNA (Figure 1A) and the movement of the corresponding machineries along the DNA enhances the torsional stress into DNA, by generating positive supercoils in front of and negative supercoils behind them (Figure 1B; Liu and Wang, 1987). At the same time, these torsional stresses considerably alter the dynamics of DNA transactions. Indeed, the DNA overwinding in front of the machineries causes a slow-down followed by an arrest of these machineries, and the DNA unwinding that produces single-stranded DNA region behind the machineries prevents DNA recognition. In addition to the topological stresses affecting the DNA supercoiling (Figure 2A), other topological constraints appear at specific locations and time points during the cellular life. For instance, the replication process generates pre-catenaned and catenated molecules: these forms must be untangled for chromosomes separation (Figure 2B). In the same way, hemicatenates are produced during the recombination and they must be removed (Figure 2B). The spatial displacements of the DNA in the cell generate knots or pseudoknots. Thus, each kind of DNA transactions generates different DNA constraints that must be solved. A class of enzymes named DNA-topoisomerases is dedicated to control the level of DNA supercoiling and solve other topological constraints. These enzymes have been discovered during the 70’s (Wang, 1971; Champoux and Dulbecco, 1972; Gellert et al., 1976).

To face the different issues due to the structure of DNA, we can postulate that DNA-topoisomerases appeared more or less within the same time period as DNA, i.e., the DNA world, because of their crucial role in the DNA metabolism.

## How do the Topoisomerases Work?

**FIGURE 3:**
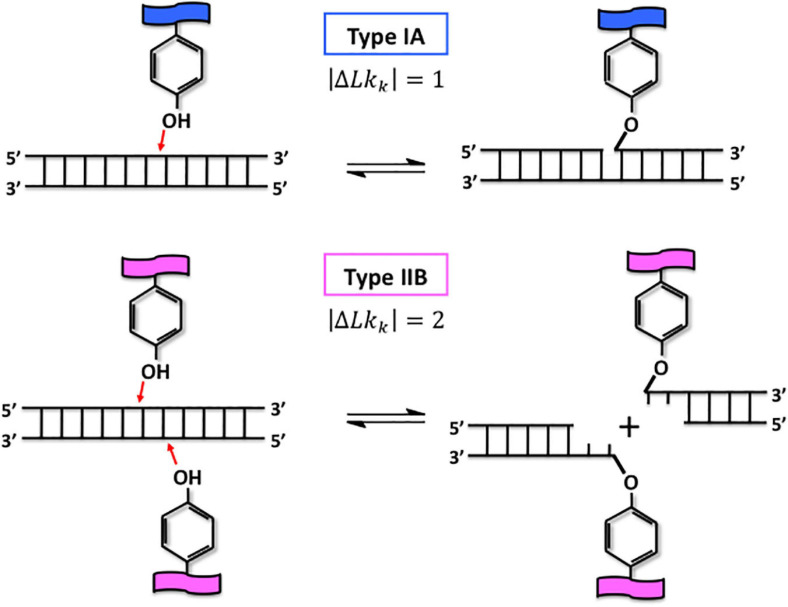
The magical power of DNA-topoisomerases: the transesterification reaction. Type IA and type IIB DNA-topoisomerases are represented by the tyrosine group, which is characteristic of their active site, in presence of double-stranded fragment of DNA (upper and lower parts, respectively). The transesterification reaction performed by type I and type II DNA-topoisomerases can be divided into three major steps. First, the tyrosine group attacks the phosphate group of the DNA backbone orientated 5’–3’, which is represented by red arrows (left part). Second, type IA, and type IIB DNA-topoisomerases transiently cleave one or two strands of DNA, respectively, by being covalently linked to the phosphate group at the 5’ extremity (right part). The double black arrow symbolizes the dynamics which exists between these two steps. The passage of one or two strands of DNA through the DNA break is performed by type I and type II DNA-topoisomerases, it occurs during the open state. After resealing the gate by reverting the reaction shown, the linking number of the DNA molecule is changed by one or two, respectively.

As written by Wang (2002), DNA-topoisomerases are true magicians of the DNA that transiently cleave one DNA strand or both and create a gate into the molecule. Consequently, another single-stranded or double-stranded DNA can pass through this gate and by this way, this passage can eliminate the torsional stress in DNA or separate postreplicative sister chromatids (Wang, 2002). To create the transient breakage within the DNA phosphodiester backbone, DNA-topoisomerases use a transesterification reaction that does not require any additional energy (Figure 3). According to their respective mechanisms, it is possible to distinguish two types of DNA-topoisomerases. The type I DNA-topoisomerases are monomeric and transiently cleave a single DNA strand while the type II enzymes form dimeric assemblies and cleave transiently the two DNA strands (Figure 3) and can decatenate DNA.

According to their types, I or II, and their own particularities, DNA-topoisomerases solve all the topological stresses present in the DNA molecules as illustrated in the Figure 2: they can remove or introduce positive and negative supercoils, decatenate and unknot DNA or remove the hemicatenates.

## Effects of Environmental Factors on the DNA

Since the end of the 70s, Carl R. Woese has proposed that the living organisms are divided into three domains, the Eukarya, the Bacteria and the Archaea (Woese et al., 1990). However, the precise root within these three domains, and especially between Eukarya and Archaea, is still actively debated (Spang et al., 2015; Da Cunha et al., 2017). Based on recent phylogenetic studies, the archaeal taxonomy becomes more and more refined and is composed of four major superphyla: TACK, DPANN, Euryarchaeota, and Asgard. The TACK superphylum includes the Thaumarchaeota, Aigarcheota, Crenarchaeota, and the Korarchaeota (Guy and Ettema, 2011; Spang et al., 2015). The DPANN superphylum comprises the Diapherotrites, Parvarchaeota, Aenigmarcheota, Nanoarcheota and the Nanohaloarcheota, and this superphylum is essentially based on candidatus taxa (Rinke et al., 2013; Adam et al., 2017; Dombrowski et al., 2019). The Euryarchaeota superphylum encompasses the Archaeoglobi, Thermococci, Diaforarchaea, and the Stenosarchaea. The Diaforarchaea comprise the Thermoplasmatales, Methanopyri, and the Methanomada (both including methanogens) while the Stenosarchaea include other methanogens and halophiles. The quite recently proposed Asgard superphylum comprises the Lokiarchaeota, Thorarchaeota, Odinarchaeota, and the Heimdallarchaeota (Spang et al., 2017). Some members of all these taxa whose genomes are fully sequenced and available are listed in Table 1.

**TABLE 1:** DNA-topoisomerases content in specific representatives of the different taxa in Archaea.

Superphyla	Rank 1 taxon	Rank 2 taxon	No. Gen.	Representative species example	Life style	Type I				Type II	
						Type IA					
						Topo III	RG	Type IB	Type IC	Type IIA	Type IIB
TACK	Crenarchaeota	Thermoprotei	95	*Pyrobaculum aerophilum*	T	1	1				1
				*Saccharolobus solfataricus*	T	1	2				1
	Thaumarchaeota	Conexivisphaeria	26	*Conexivisphaera calida*	T	1	1*				1
		Nitrososphaeria		*Nitrososphaera viennensis*	M	1		1			1
	Korarchaeota		1	*Cand. Korarchaeum cryptofilum*	T	1	1				1
DPANN	Nanoarchaeota	Nanoarchaeales	2	*Nanoarchaeum equitans*	T	1	1				1
	unclassified		1	LC1Nh	?	1					1
Euryarchaeota	Archaeoglobi		8	*Archaeoglobus profundus*	B, T	1	1			1(G)	1
	Thermococci		44	*Pyrococcus abyssi*	B, T	1	1				1
	Diaforarchaea	*Aciduliprofundum*	15	*Aciduliprofundum boonei*	T	1	1			1(G)	1
		Thermoplasmata	18	*Thermoplasma volcanium*	T	1				1(G)	1
				*Methanomassiliicoccaceae archaeon*	M	1				1(G)	1
	Methanopyri		1	*Methanopyrus kandleri*	T	1	1		1		1
	Methanomada	Methanobacteria	32	*Methanothermus fervidus*	T	1	1			1	1
		Methanococci	21	*Methanotorris igneus*	T	1	1			1	1
	Stenosarchaea	Methanomicrobia	54	*Methanosarcina mazei*	M	2				1	1(+)
		Halobacteria	92	*Halobacterium salinarum*	H	1				1(G)	1(+)
Asgard	Cand. lokiarchaeota		1	*Cand. Prometheoarchaeum syntrophicum*	M	1				1(G)	1

The taxonomy used in this table are from Lifemap^1^ (de Vienne, 2016). Only species representative of a taxon and with a completely sequenced genome have been considered. The methanogens belonging to different taxa are indicated in blue. No. Gen. indicates the number of available sequenced genomes corresponding to the taxa of the rank 1 or 2. Cand. is for Candidatus. The number of each topoisomerase encoding gene found has been indicated. RG indicates the presence of one or two reverse gyrase encoding genes (in red) and (*) is to pinpoint that several Candidatus species of thermophilic Thaumarcheota do not possess a reverse gyrase encoding gene. (G) indicates the presence of a putative gyrase as type IIA topoisomerase (in magenta). The Type IIB present in all archaea corresponds to Topo VIs, but some archaeal species exhibit an additional type IIB, either a Topo VIII or a Mini-A, which is noted (+). The abbreviations used for the life style are as follows: mesophily (M), thermo or hyperthermophily (T), barophily (B), halophily (H) or unknown (?). To check the presence of the different DNA-topoisomerases, we used TBlastN with the non-redundant NCBI nucleotide collection until April 2021.

Given the extent of the growing archaeal diversity, the Archaea are like a gold mine: they give us the opportunity to spotlight a significant set of new subfamilies of topoisomerases allowing a better understanding of the global phylogeny of topoisomerases and to precise their respective roles. They also give us a new insight in understanding the DNA stress management occurring during all DNA processes (see Table 1, and the corresponding comments in the see section “DNA-Topoisomerases Content and Their Respective Activities in Archaea”).

Amongst the Archaea, a certain number of them live in a moderate environment and must face the same issues as ambient bacteria and eukaryotes, while others referred as extremophiles live in unusual environments, at the frontiers of conditions enabling life. Hence, extremophiles live at a very high/low pH, a salt concentration close to the limit of solubility, a very high pressure and/or very high temperature, conditions that may impact the macromolecules stability (Dance, 2020). As a consequence, these organisms possess particular adaptations to deal with these unusual environments and keep the functionality and the stability of their genome.

DNA structure, and particularly the DNA helical repeat, is very sensitive to the variations of numerous physico-chemical parameters. DNA is therefore a very efficient molecular probe for sensing a variety of signals. In a topologically constrained DNA, i.e., a covalently closed circular DNA or a DNA with its ends that are not free to rotate like a loop, an apparently very limited change in the helical repeat promotes a significant change of the supercoiling level, also referred as the writhe (Wr). This is the consequence of the distribution of the topological constraints on two geometrical contributions, the DNA twist (Tw) that depends on the DNA helical repeat, and the writhe of DNA axis as it is summarized in the following topological equation:

$$L_{k}=T_{w}+W_{r}$$

where

$$T_{w}=N/h$$

and Lk is the linking number, the number of links between the two DNA strands, N is the number of base pairs, h is the mean of the helical repeat of the DNA and Wr is the curvature of the DNA axis, the DNA supercoiling or writhe (Wang, 2009).

The meaning of this equation is the following: if the helical repeat of the DNA or the supercoiling is modified, the other geometrical parameter is immediately and proportionally changed, as long as no topoisomerase changes the linking number. For example, when the helical repeat of the DNA is modified by changing the salt concentration or the temperature, an appropriate DNA supercoiling of the whole DNA molecule is triggered. A similar adjustment has to be performed when DNA melting occurs in a particular region. Thus, a faint global change or a significant local change can promote an adaptation of the topological state of the whole genome, even at a long distance. This crucial DNA property can have particularly important consequences for the extremophiles.

In halophiles, the intracellular salt concentration can reach molar concentrations of NaCl or KCl, with an additional concentration of MgCl_2_ up to 0.05 M. However, experimental data about DNA structure are available only for a low salt concentration range. In the absence, or at a very low concentration of MgCl_2_, increasing NaCl concentrations, up to 0.2 M, decrease monotonously the helical repeat of the DNA. At higher MgCl_2_ concentrations, up to 50 mM, the effect of increasing NaCl concentrations can be neglected (Rybenkov et al., 1997). Therefore, high concentrations of NaCl are counterbalanced by high MgCl_2_ concentrations in halophiles. The hydration is lower in all the macromolecules and especially DNA. The high salt concentration favors the DNA structural transitions, in particular the transition from B to Z. However, it seems reasonable to consider that these high salt concentrations might affect only slightly the DNA structure *in vivo* because of the DNA protection with the interaction of DNA-binding proteins limiting the access of salt-derived cations. Thus, no specific adaptation of DNA is clearly required. Moreover, it is noteworthy that the GC content of these organisms is very high but this bias in DNA composition rather reflects the requirement for Asp, Glu, Thr, and Val amino acids in halophile proteins (Paul et al., 2008; Takahashi et al., 2018).

The DNA helical repeat is very sensitive to pH concentrations, essentially when it is below 5 or above 9 units. Although acidophiles, alkaliphiles or halophiles live at extreme pH, their intracellular pH value is close to neutrality. Consequently, pH does not seem to be a parameter to be considered for both the structure and metabolism of DNA in Archaea.

Some archaeal species live at very high pressure and it is known that DNA supports very well this condition (Girard et al., 2007). However, some structural changes in a B-DNA helix occur with hydrostatic pressure. Indeed, the inner DNA cavity is reduced by decreasing both the DNA hydration and the base-pair spacing (referred as the rise of the DNA), upon increasing hydrostatic pressure (Wilton et al., 2008). This effect is limited and it is also the case for the modification of the DNA twist. Consequently, the DNA base pairing is only slightly modified and pressure has limited consequence on the DNA structure and topology.

Finally, the most important issue for DNA is the high temperature at which the thermophiles and hyperthermophiles live, i.e., sometimes near the DNA melting temperature. All the chemical processes increase their rate with increasing temperature, and it is particularly the case for the processes that lead to natural DNA damages such as deaminations, oxidations, alkylations, abasic site formations or phosphodiester bond breakages (Marguet and Forterre, 1994). All these modifications are dramatically enhanced with increasing temperature (Lindahl, 1993; Jacobs and Grogan, 1997). Nevertheless, covalently closed circular DNA resists to temperature as high as 107°C in the presence of salt (Marguet and Forterre, 1998). However, some of DNA modifications are increased in single-stranded portions of DNA, and a snowball effect of the temperature can lead to an increase of double strands breaks which corresponds to the worst DNA damage because they impair the genetic information. The increase of all these DNA damages could lead to a higher mutation frequency in the thermophilic organisms but it is not the case actually, indicating that thermophilic organisms exhibit highly coordinated DNA repair pathways (Jacobs and Grogan, 1997; Reilly and Grogan, 2002). In addition to the intrinsic high thermostability of the DNA repair proteins, the different repair pathways involved are, for most of them, permanently expressed in a highly coordinated way, leading to prevent the mutagenetic effect of the high temperature (Gerard et al., 2001; Grogan, 2015; Larmony et al., 2015). However, a key role for the post-translational protein modifications was reported in response to DNA damages (Kish et al., 2016). In addition to the DNA damages, DNA structure itself is highly sensitive to the temperature, in particular the helical repeat of the DNA increases with the temperature by 0.0105°×°C^–1^ × bp^–1^ (Jaxel et al., 1989; Duguet, 1993). This apparent small effect has in fact a considerable impact on the DNA topology and even on the tridimensional structure of the entire genome. Moreover, as illustrated in Figure 1, the dynamics of the DNA transaction processes modify the DNA topology locally which in turn could destabilize either part or the whole genome. Additionally, the transient opening of the DNA, known as the DNA breathing, occurs below the melting temperature. It is due to the thermal fluctuations, and both frequency and expansion of the DNA breathing increase with (i) the temperature, (ii) the DNA tension and, (iii) the DNA underwinding, as it was recently illustrated by using magnetic tweezers (Bizard et al., 2018). Indeed, to face the effects of the high temperature, all thermophiles and hyperthermophiles from both archaea and bacteria possess a particular topoisomerase, the reverse gyrase. This enzyme is able to remove very efficiently negative supercoils and limits the DNA breathing by introducing positive supercoiling. This decrease of single-stranded DNA formation was evidenced by the inhibition of the inversion reaction, catalyzed by the Hin inverstase, by overwinding DNA (Lim et al., 1997). Moreover, during the transcription initiation process, the positive supercoiling inhibits the open complex formation at moderate temperature (48°C) but not at high temperature, i.e., 75°C (Bell et al., 1998). This highlights that positive supercoiling limits the DNA breathing and consequently controls the DNA melting. This property can explain the presence of reverse gyrase as a prerequisite for the life at high temperature, and it is the reason why reverse gyrase is considered the molecular marker of the thermophily as it was proposed (Forterre, 2002).

If the physico-chemical environment acts undoubtly on DNA structure, it is important to keep in mind that cellular components, as the proteins that shape DNA, can limit some deleterious effects of the temperature.

## DNA-Topoisomerases Content and Their Respective Activities in Archaea

As for all the bacterial and eukaryotic living organisms, Archaea possess the two types of DNA-topoisomerases, at least one of each type. The detail of the topoisomerases content in representative species of different archaeal groups is summarized in Table 1.

## Type II DNA-Topoisomerases

**FIGURE 4:**
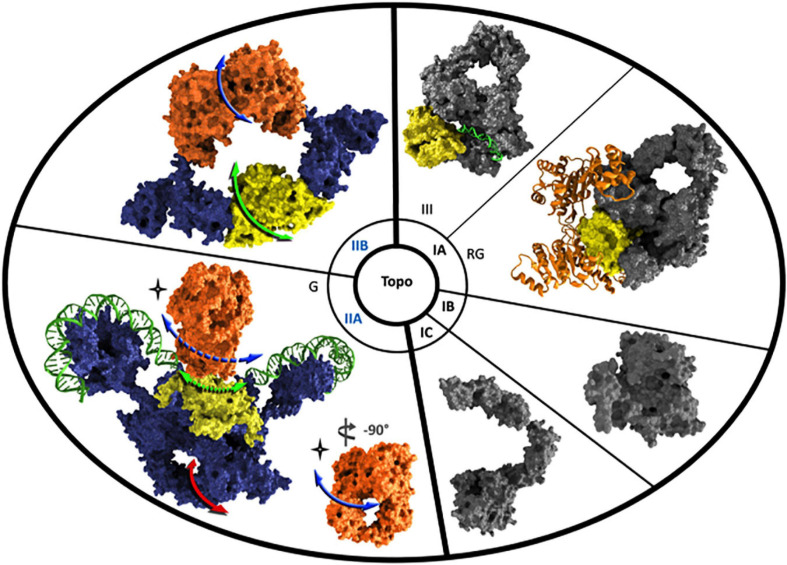
Structural schematic view of the different types of DNA-topoisomerases. The 3-D structures of representative type I and type II DNA-topoisomerases are shown. Key domains such as ATPase domain and Toprim domain are highlighted in orange and yellow, respectively. SF2 helicase-ATPase domain in reverse gyrase is represented by an orange ribbon. DNA molecules are colored in green. Topo IIA is exemplified by gyrase and DNA is all around the ß-pinwheel structure and through the DNA-gate (green arrows) located within the Toprim domain. A perpendicular view of ATP-binding domain allows to show the ATP-gate (blue arrows) as a hole in comparison with the C-gate (red arrow) hole visible in the lower part of the carboxy-terminal domain in blue. The full and dashed arrows outline the direct and indirect visualization of the DNA position during the passage through the DNA-gate, respectively. The 3-D structures have been visualized using Visual Molecular Dynamics and rendering with 3DS MAX. The PDB numbers used for these structures are 3PX7 (Zhang et al., 2011), 4DDU (Rodríguez and Stock, 2002), 3M4A (Patel et al., 2010), 5HM5 (Rajan et al., 2016), 6RKW (Vanden Broeck et al., 2019), and 2Q2E (Corbett et al., 2007), and they refer to Topo III (noted as III), reverse gyrase (noted as RG), Topo IB, Topo IC, Gyrase (noted as G), and Topo IIB, respectively.

Type II DNA-topoisomerases are divided into two subfamilies: types IIA, IIB. The proteins of these two subfamilies exhibit a homodimeric (α_2_) or heterotetrameric (α_2_ß_2_) symetric structure with some protein domains in common but their global organizations are different. Briefly, the domain that contains the tyrosine responsible for the transesterification reaction is always located in the carboxy-terminal part for both types. The ATP-binding domain, so-called the Bergerat fold, is present in the amino-terminal part of the proteins while the Toprim domain is present in the amino-terminal part for the Topo IIA and in the carboxy-terminal part for the Topo IIB (Figure 4). Each of them exhibits specific additional domains (Graille et al., 2008). These structural organizations lead to the presence of only one hole delimited by two contacts that form two gates for the Topo IIB, the ATP-gate and the DNA-gate that correspond to the catalytic tyrosine and the Toprim domain. In contrast, two holes are delimited by three contacts forming three gates for the Topo IIA, the ATP-gate, the DNA-gate and the additional C-gate (Figure 4). Amongst the Topo IIA, the DNA gyrase is able to introduce negative supercoils into DNA. This is the consequence of the DNA conformation on the carboxy-terminal domain that forms a ß-pinwheel structure containing motifs named GyrA-box (Vanden Broeck et al., 2019; Figure 4).

In terms of type II DNA-topoisomerases content, all archaeal genomes sequenced so far, are characterized by the presence of one gene belonging to the type IIB subfamily (Table 1; Forterre et al., 2007; Graille et al., 2008). This subfamily comprises three members, the Topo VI, the Topo VIII and the Mini-A (Takahashi et al., 2020). The Topo VIs are heterotetrameric proteins essentially present in archaea, but also found in bacteria and eukaryotes. In eukaryotes, the Topo VI-like acts during the meiosis (Robert et al., 2016; Vrielynck et al., 2016) and in adddition during the endoreduplication in plants (Vrielynck et al., 2016). Unfortunately, very few information about the activity of these enzymes has been reported and most of these studies concern the enzymes from the *Sulfolobus* and *Saccharolobus* genera. Thus, from a biochemical point of view, the particularity of the Topo VI is to have two base pairs between the two cleavage sites (Figure 3) instead of four base pairs for the type IIA enzymes (Schoeffler and Berger, 2008). It was recently reported that the Topo VI from *Saccharolobus shibatae* (previously named *Sulfolobus shibatae*) is able to relax negatively or positively supercoiled DNA with a preference for the positive supercoils, but is not very efficient at decatenating DNA (Couturier et al., 2019). Finally, these enzymes are sensitive to the drugs that interfere with the ATPase activity, like radicicol that is a powerful Topo VI topoisomerases inhibitor (Gadelle et al., 2005). The Topo VIII differs from the Topo VI by a short carboxy terminal extension, and most of them are homodimeric proteins. The Mini-A is closed to Topo VIII with some differences especially in some motifs and in the carboxy terminal domain. Topo VIII and Mini-A are mainly encoded by mobile genetic elements and only few archaeal strains possess these genes (Takahashi et al., 2020). Finally, a very preliminary study showed faint activities with significant differences between the three bacterial enzymes tested (Gadelle et al., 2014). To date, no characterization of Topo VIII or Mini-A from archaeal species has been reported, preventing any conclusion on their role(s) within the archaeal cells.

In addition to the type IIB topoisomerase, a type IIA topoisomerase is usually present in the Euryarchaeota except the *Methanopyrus kandleri* and the Thermococci (Table 1). Moreover, several type IIA topoisomerases exhibit GyrA-box motifs within their carboxy-terminal domain. As mentioned above, these motifs present in the ß-pinwheel structure represent a hallmark of a gyrase protein (Schoeffler and Berger, 2008). It is the case for the halophiles, the Diaforarchaea and the Archaeoglobi (Klenk et al., 1997) suggesting a gyrase activity in these organisms as in the Candidatus Lokiarchaeota (Table 1).

## Type I DNA-Topoisomerases

Type I DNA-topoisomerases are divided into three subfamilies that are not related: the types IA, IB and IC. Basically, all the type I DNA-topoisomerases are able to relax supercoiled DNA, either negatively or positively supercoiled, but, due to their requirement for single-stranded DNA, the type IA is inefficient on positively supercoiled DNA (Figure 2A). Based on their sequences and domains composition and their activities, it is possible to distinguish three subfamilies among the type IA: the Topo I, the Topo III and the reverse gyrase (Figure 4). All the living cells exhibit at least one type IA apparently belonging to the Topo III subfamily (Garnier et al., 2018 and unpublished results, HD and MN). Interestingly, most of these enzymes possess an RNA-topoisomerase activity which appears important to untangle long RNA that form pseudoknots (Figure 2C; Adam et al., 2016). It was hypothesized that this RNA-topoisomerase activity could be crucial in the RNA world, suggesting that the type IA is one of the most ancient enzymes (Adam et al., 2016; Garnier et al., 2018). Interestingly, the Methanomicrobia possess two type IA, probably belonging to the Topo III subfamily but further studies are needed to confirm this classification. The type IB is mostly present in eukaryotes while type IC is present in only one archaeon, *M. kandleri* (Table 1). This unique location was interpreted as a probable acquisition of a recombinase gene recently transferred from a virus (Forterre and Gadelle, 2009). This recombinase is able to perform the transesterification reaction as numerous recombinases and thus can act as a topoisomerase (Rajan et al., 2016).

All the Archaea, as all Bacteria and Eukarya, and regardless of their life style, have at least one type IA enzyme which is a Topo III (Table 1). This essential enzyme is known to be involved in the genome stability in both Bacteria and Eukarya (Chen et al., 2013). In Archaea, this enzyme was first described in *Solfataricus solfataricus* (Dai et al., 2003). The precise analysis of its activity showed that this enzyme poorly relaxes negatively supercoiled DNA but is very efficient at decatenating single- or double-stranded DNA (Bizard et al., 2011, 2018).

In the course of the discovery of the DNA-topoisomerases in hyperthermophilic archaea, a completely new topoisomerase was discovered as early as 1984. It is an enzyme that overwinds DNA, i.e., that is able to positively supercoil the DNA (Figure 2A; Nadal, 2007). This new enzyme was named reverse gyrase (Kikuchi and Asai, 1984). Surprisingly, we have shown that this overwinding activity is carried out by an ATP-dependent type I topoisomerase (Forterre et al., 1985; Nadal et al., 1988). Some years later, we evidenced that this amazing DNA-topoisomerase results from the fusion of two domains, one that corresponds to a classical topoisomerase IA and the other to an ATPase that is related to the SF2 helicase (Figure 4; Confalonieri et al., 1993; Jaxel et al., 1996). As early as 1990, it was shown that reverse gyrase is present in all hyperthermophilic archaea (Bouthier de la Tour et al., 1990; Table 1), but also in hyperthermophilic bacteria (Bouthier de la Tour et al., 1991). It is noteworthy that the DNA-binding protein Sso7d is able to constrain negative supercoils of DNA and consequently inhibits reverse gyrase (Napoli et al., 2002). This inhibition might be the consequence of the decrease of the unwinding capability of DNA, which is required for the activity of the reverse gyrase, as we evidenced previously (Yang et al., 2020).

Most Crenarchaeota have two reverse gyrases, TopR1 and TopR2 (Table 1), and we have shown that the two enzymes do not exhibit the same biochemical properties (Bizard et al., 2011). *In vitro*, TopR1 activity is gradually enhanced in response to the increasing temperature while TopR2 is a highly processive enzyme regardless of the temperature. *In vivo*, when *S. solfataricus* (previously named *S. solfataricus*) are maintained for a long time at low temperature, TopR1 disappears while the amount of TopR2 enzyme remains unchanged (Couturier et al., 2014). These results suggest a sub-functionalization displayed by the two reverse gyrases (Garnier et al., 2018). A recent phylogenetic analysis confirms this hypothesis (Catchpole and Forterre, 2019). Taking advantage of the properties of TopR2, we recently deciphered, by using magnetic tweezers, the first steps of the reverse gyrase reaction. Briefly, TopR2 binding induces a DNA unwinding about 20 base pairs. After ATP-binding, this unwinding decreased to 10 base pairs, reflecting a conformational change into the protein that probably induces a particular shape to the DNA before the cleavage and the strand passage reaction. It is this particular DNA conformation that leads to an increase of the linking number by one after the DNA resealing (Yang et al., 2020). Even though nearly all reverse gyrases have a monomeric structure with two domains, it is noteworthy that in *Nanoarchaeum equitans*, the two domains of the reverse gyrase are naturally split into two polypeptides (Capp et al., 2010).

Since the reverse gyrase is present in all the hyperthermophiles except the *Thermoplamata* genus that has no reverse gyrase encoding gene (Table 1), we now consider that reverse gyrase is the marker of the thermophily (Forterre, 2002). In the case of Thermoplasmatales, the absence of reverse gyrase could be due to the moderate temperature at which these organisms live and/or due to the DNA stabilization by (i) the classical archaeal histone-like Alba proteins and (ii) the wrapping of DNA around the HU-like protein HTa which is specific to the Thermoplasmatales (Stein and Searcy, 1978; Maruyama et al., 2020).

Even if the type IB DNA-topoisomerase was considered a specificity of the Eukarya, it was recently shown that a Topo IB encoding gene is present in some viruses, bacteria and archaea. The basic relaxation and cleavage activities have been shown for some of these species (Dahmane et al., 2016) and in particular in mesophilic members of Thaumarchaeota such as *Nitrososphaera viennensis* (Table 1; Forterre et al., 2007). As expected, a reverse gyrase encoding gene is present in thermophilic members of Thaumarchaeota (see *Conexivisphaera calida* in Table 1), as in the other thermophilic organisms. However, a recent genomic analysis indicates that several members of thermophilic Thaumarcheota of the genus *Nitrosocaldus* such as *Candidatus Nitrosocaldus cavascurensis* do not possess a reverse gyrase encoding gene (Abby et al., 2018).

*Methanopyrus kandleri* stands out once again from other archaeal members, as it exhibits a reverse gyrase split into two polypeptides whose the respective delimitations do not overlap with each of the two domains, the helicase and topoisomerase ones, and with an additional domain in the topoisomerase part (Krah et al., 1996). Finally, it also possesses the completely new and atypical topoisomerase Topo IC (Table 1 and Figure 4; Taneja et al., 2006).

## DNA Supercoiling in Archaea

We have seen that the Archaea possess a topoisomerases content that differs from those of the mesophilic bacteria or the Eukarya, and this raises the question of the DNA topology and supercoiling in Archaea. Unfortunately, very few information is available, but we have summarized the different topoisomerase activities in the Figure 2.

In the course of the determination of the DNA supercoiling *in vivo*, the superhelical density of a set of plasmids issued from different archaea was determined (Charbonnier and Forterre, 1994; López-García and Forterre, 1997). The plasmids of the extreme halophiles appear slightly more negatively supercoiled than the mesophilic bacteria, and the salt concentration of the medium has only a small effect on DNA supercoiling of the plasmids (Charbonnier and Forterre, 1994; Mojica et al., 1994). For mesophilic methanogens, the plasmid supercoiling is in the same range as the mesophilic bacteria. Hence, it appears there is no obvious supercoiling adaptation for the mesophilic halophiles and methanogens. However, the superhelical density of the plasmids from thermophilic methanogens is more or less relaxed, and an increase of the growth temperature of the halophiles leads to a decrease of the amount of negative supercoils (Charbonnier and Forterre, 1994; Mojica et al., 1994; López-García and Forterre, 1997). This reflects an important effect of the temperature on DNA supercoiling *in vivo* due to the intrinsic sensitivity of the DNA helical repeat in response to temperature variations as discussed above.

The presence of reverse gyrase in hyperthermophilic archaea had immediately raised the question of the DNA supercoiling in the hyperthermophiles. Thanks to the discovery of the temperate virus SSV1 in *S. shibatae* (Martin et al., 1984), we have shown that its DNA is highly positively supercoiled in the viral particle while a wide range of viral DNA supercoiling, spanning from moderately negatively supercoiled to highly positively supercoiled, is observed during the multiplication step in the cell (Nadal et al., 1986). This important result indicates that i) positively supercoiled DNA exists *in vivo*, and ii) reverse gyrase is able to positively supercoil DNA *in vivo* as it was evidenced *in vitro*. The analysis of a set of plasmids isolated from hyperthermophiles showed that they are relaxed or slightly positively supercoiled. When the hyperthermophilic barophiles are cultivated at ambient pressure, their DNA supercoiling is within the same range than those observed in the other hyperthermophiles, i.e., relaxed or slightly positively supercoiled (Charbonnier and Forterre, 1994; López-García and Forterre, 1997). However, the plasmid pGS5 isolated from *Archaeoglobus profundus* is negatively supercoiled (López-García et al., 2000). This archaeon, isolated from deep-sea thermal vents, contains both a reverse gyrase and a gyrase (Table 1) suggesting that in the laboratory conditions used, gyrase activity might be more efficient than the reverse gyrase activity. Thus, it appears that it is the high temperature that promotes positive DNA supercoiling and prevents negative supercoiling. This crucial adaptation limits the DNA melting as it was shown during the transcription (Bell et al., 1998).

All the living organisms require a fine tuning of their DNA topology. To get some information about this regulation in Archaea, variations of both plasmidic DNA supercoiling or DNA-topoisomerases content were quantified. It was shown that halophiles are sensitive to inhibitors of the bacterial or eukaryal type IIA DNA-topoisomerases. Both novobiocin and coumermycin target the ATPase site of the gyrase. It was shown that the presence of novobiocin induced an increase in the plasmidic DNA superhelical density, giving positively supercoiled plasmids in halophiles as well as in *Escherichia coli* (Sioud et al., 1988). This indicates that in halophiles, the DNA supercoiling is essentially controlled by the DNA gyrase. Moreover, the presence of novobiocin in the growth medium does not induce a cell filamentation, which is a phenotype characteristic of a direct or indirect inhibition of the chromosome decatenation blocking the chromosome segregation and consequently the cell division (Forterre et al., 1986). This effect can be attributed to the gyrase due to mutations in the corresponding genes leading to a resistance to this antibiotic (Holmes and Dyall-Smith, 1991). Other inhibitors, such as the fluoroquinolone or etoposide, stabilize the cleaved complex form of the bacterial or eukaryal type IIA topoisomerase, respectively (Forterre et al., 1986). The presence of etoposide in the growth medium leads to a cell filamentation and increases the amount of protein-DNA covalent complexes. Thus, it is tempting to attribute this increase to the formation of a cleaved complex involving a type II topoisomerase. As we have seen, halophiles possess both a type IIA related to gyrase and a type IIB topoisomerase. However, except for the novobiocin, we have no information about the sensitivity of each type II enzyme to the other inhibitors. Consequently, it is difficult to attribute a precise role to each type II enzyme in these experiments.

The regulation of the DNA topology in hyperthermophiles was studied during cell growth, at different temperatures or upon a temperature change. Even if the DNA supercoiling state is not exactly the same in the Thermococcales and Sulfolobales, an increasing temperature leads in both cases to an overwinding of DNA. This clearly indicates that positive supercoiling is a response adapted to the high temperature (López-García and Forterre, 1997). We have shown that the mRNA level of *topR1* is down-regulated with high temperatures compared to the *topR2* mRNA level remaining unchanged (Garnier and Nadal, 2008). More recently, we have demonstrated that TopR1 is the key enzyme responsible for the homeostatic control of the DNA supercoiling in *S. solfataricus* (Couturier et al., 2019). The protein level of TopR2 and Topo VI remains constant and their respective activities are strongly inhibited at high temperatures (Couturier et al., 2014, 2019). TopR2 is not involved in this regulation while TopoVI assumes the removal of the excess of positive supercoils when necessary. TopA is not either involved in the homeostatic control of the DNA supercoiling because both *topA* mRNA and TopoA protein amounts remain very low or even undetectable in *Saccharolobus* in all the conditions tested (Garnier and Nadal, 2008; Couturier et al., 2014, 2019). Nevertheless, TopA could help or even replace TopoVI to decatenate DNA as it is still very efficient at unknotting or decatenating DNA at high temperature (Bizard et al., 2018).

## Genetics of the DNA-Topoisomerases in Archaea

Genetics is very efficient at assigning a precise role to a gene in an organism. Unfortunately, only few genetics information is available concerning the DNA-topoisomerases in Archaea. Except the mutant resistant to the novobiocin in halophiles, which provides clue about the role of the gyrase in this organism (Holmes and Dyall-Smith, 1991), the genetic studies concern the type IA and essentially the reverse gyrase. In *Thermococcus kodakarensis*, the reverse gyrase gene was deleted and the consequence was a decrease of the growth rate at high temperature (Atomi et al., 2004). Thus, the presence of reverse gyrase was interpreted as not essential for the growth at relatively high temperature. However, a similar analysis in *Pyrococcus abyssi*, another archaeon belonging to the Thermococci subgroup of the Euryarchaeota, showed that the reverse gyrase gene clearly turns essential at very high temperature (Lipscomb et al., 2017). In *Sulfolobus islandicus*, the deletion of *topR1* and *topR2* genes has been first reported as lethal, suggesting that both genes are essential (Zhang et al., 2013). However, more recently, it has been shown that it is possible to disrupt *topR1* or *topR2* genes separately, but the obtention of the corresponding mutants was possible only after a very long incubation time of the transformation plate, up to 3 weeks for the *topR2* mutant (Zhang et al., 2018). This underlines the importance of the two reverse gyrase genes in Sulfolobales. Moreover, the viability of *topR2* mutant is largely reduced indicating that *topR2* is very important *per se* and even more important than *topR1*. Unfortunately, the presence of compensatory mutations selected in these mutants has not been reported. These data point out again that both genes do not have the same role in the Sulfolobales as we previously proposed (Bizard et al., 2011; Couturier et al., 2014, 2019).

Besides the deletion of the reverse gyrases encoding genes, a deletion of *topA*, the Topo III encoding gene, was obtained in *S. islandicus*. This strain grows more slowly than the wild-type and a defect in coupling the genome segregation with the cell division was observed (Li et al., 2011). This is consistent with the decatenation activity of the Topo III further published (Bizard et al., 2018; Couturier et al., 2019).

## Involvement of DNA Topoisomerases in DNA Repair and Genome Stability

As discussed above, DNA-topoisomerases solve, in all the living organisms, topological stresses directly created by DNA-based machineries (Figure 1) and, in this way, make sure DNA replication, repair and recombination occur properly. Type IA topoisomerases are actually directly involved in homologous recombination, at least when SF2 helicases are implied in corresponding specific steps (Chen et al., 2013). In Archaea, the involvement of the type II topoisomerases in DNA repair has not been reported yet and most data concern once again the reverse gyrase. Since positive supercoiling prevents single-stranded DNA formation, and considering that single-stranded DNA is more sensitive in DNA damages, reverse gyrase participates to DNA repair. In addition, reverse gyrase exhibits at stoichiometric amount a heat-protective DNA chaperone activity (Kampmann and Stock, 2004). A direct involvement of reverse gyrase in DNA repair pathway has been shown. Indeed, in response to UV irradiations, reverse gyrase is recruited on DNA forming a stable covalent complex (Napoli et al., 2004b). When the DNA is damaged by alkylating agent, the reverse gyrase is degraded by a specific metal-dependent protease. Within the same time, a degradation of the genome occurs (Valenti et al., 2006). Recently, it was reported that TopR1 could have a protecting effect on the genome degradation upon MMS treatment (Han et al., 2017). Single-strand binding protein (SSB) is a key protein involved in the DNA repair and recombination processes. It was shown that the reverse gyrase activity is stimulated by SSB protein supporting a direct role of reverse gyrase in DNA repair and recombination processes (Napoli et al., 2004a). Moreover, reverse gyrase is able to form an additional direct interaction with PolY, the translational DNA polymerase. If PolY does not modify the TopR1 activity *in vitro*, TopR1 inhibits the polymerase reaction and the presence of both helicase and topoisomerase domains of the protein is required to achieve this inhibition (Valenti et al., 2009). Moreover, the presence of SSB protein, once again, strengthens this inhibition. Hence, by inhibiting the translational polymerase, reverse gyrase prevents mutagenesis and contributes to genome stability. Finally, in response to alkylating agent, PolY is probably degraded by the same metal-dependent protease previously mentioned for reverse gyrase disappearance (Valenti et al., 2009). All these data clearly highlight an important role of reverse gyrase in DNA repair in Sulfolobales (Vettone et al., 2014). The involvement of reverse gyrase in the response to alkylating agent occurs also in *P. abyssi*. In this organism, the unique reverse gyrase interacts with the DNA glycosylases AlkA and OGG1 and the single-strand binding protein RPA (Hausner et al., 2013). This function of reverse gyrase in DNA repair is strengthened by its interaction with DNA2 and Rad25/XPB, two proteins involved in DNA repair (Hausner et al., 2013). The network of key DNA repair proteins that interact physically and functionally with reverse gyrase clearly highlight once again its role in DNA repair pathways. Finally, reverse gyrase is able to unwind synthetic Holliday junction and promotes annealing of oligonucleotide indicating its direct involvement in DNA recombination process and its implication in genome stability (Jamroze et al., 2014).

In most Crenarchaeota, the presence of two reverse gyrases makes it difficult to assign the precise role for each reverse gyrase in the different works reported, and in particular for the TopR2. However, taking into account that (i) TopR2 is a highly processive enzyme (Bizard et al., 2011), (ii) TopR2 does not response to the temperature variations both in terms of activity and gene expression (Garnier and Nadal, 2008; Couturier et al., 2014), (iii) TopR2 is clearly not involved in the homeostatic control of DNA supercoiling (Couturier et al., 2019), and iv) the *topR2* expression profile exhibits an increase during G1-S transition phase of the cell cycle (Hjort and Bernander, 2001), we favor an involvement of TopR2 in the replication and DNA repair pathways. However we cannot exclude that TopR1 could be involved in these processes in particular conditions. The tight coupling existing in reverse gyrase, and more particularly in TopR2, between the RecQ-like and topoisomerase domains also suggests a role in homologous recombination. In both Eukarya and Bacteria, the resolution of hemicatenanes required a Topo III in association with a SF2 helicase belonging to the RecQ family such as the complex Topo III-Sgs1 in yeasts (Gangloff et al., 1994), Topo III-RecQ in *E. coli* (Harmon et al., 1999) or Topo IIIα with BLM, WRN or RecQ5α (Wu et al., 2000; Mankouri and Hickson, 2007). In *S. solfataricus*, we have reported that Topo III alone is able to solve hemicatenated or catenated DNA (Bizard et al., 2018). Moreover, Topo III interacts with the SF2 helicase Hel112 and modulates its activity in different ways, depending on the substrate used (Valenti et al., 2012). This functional interaction is a reminiscence of the universal SF2-Topo III associations that are essential, at least, during homologous recombination. It was reported that hyperthermophiles are able to face an accumulation of double strand breaks (Gerard et al., 2001; Larmony et al., 2015). Indeed, the repair of such DNA breakage involves the homologous recombination pathway (Quaiser et al., 2008; She et al., 2009; Kish et al., 2016). However, these SF2-Topo III complexes could be required to solve toxic structure in many other DNA processes such as D-loop which can be produced as a DNA intermediate during DNA metabolism, as recently suggested (Hogrel et al., 2020).

It is noteworthy that three different type IA topoisomerase-SF2 helicase associations co-exist in *S. solfataricus*. Two of them occur within each of the two reverse gyrase proteins, the interaction being within the same polypeptide. The third one involves two physically independent polypeptides, Topo III and Hel112, similarly to what happens in Eukarya and Bacteria. The redundancy of such an association between a SF2 helicase and a type IA topoisomerase probably reflects the multiplicity of the critical situations that organisms must cope within a very short time, and this underlines the importance of these enzymes for the genome stability in a critical environment.

Finally, RNA topoisomerase activity of Topo III raises the question of a possible role in RNA metabolism to solve RNA knots or avoid R-loop formation, these new questions remain to be addressed in Archaea (Garnier et al., 2018). Moreover, a high frequency of putative G-quadruplex sequences that has been recently observed in extremophilic archaea, points out a putative role of this particular DNA structure in these organisms (Brázda et al., 2020).

## Concluding Remarks

To date, in spite of their essential role, it is obvious that only few information about topoisomerases in Archaea is available yet. Currently, most studies focus on hyperthermophiles, and especially on type IA topoisomerases. The essential role of the different type IA topoisomerases in DNA metabolism has been highlighted by both *in vitro* and *in vivo* experiments. Beside their role in DNA supercoiling, the apparent redundancy of the association existing between SF2 helicases and type IA DNA-topoisomerases highlights the multiple role(s) of these proteins in DNA repair and genome stability in most Crenarchaeota. However, further studies are needed to decipher the precise role of these differents topoisomerases in Archaea.

More generally, it is important to underline that studies carried on DNA-topoisomerases from Archaea largely contribute to the global knwoledges about the understanding of such enzymes and the corresponding fields of investigation. For instance, the discovery of a completely new type IC DNA-topoisomerase in only one archaeon raises the question about the domestication of the recombinases as DNA-topoisomerases. The positive supercoiling activity of the reverse gyrase and the existence of positive supercoiled DNA *in vivo* helped us understand the importance of positive supercoiling in all the living organisms and also the relevance of the helicase-topoisomerase crosstalk. The discovery of the Topo VI is also a real breakthrough not only by adding a new family in the topoisomerase diversity but also by showing that Spo11, a protein involved in the initiation of the meiosis, groups with this new topoisomerase family. Quite recently, the structure of *S. shibatae* Topo VI has allowed to find the ATPase subunit of this special topoisomerase in Eukarya (Robert et al., 2016; Vrielynck et al., 2016). Hence, Archaea are key organisms to decipher the meiotic recombination in the Eukarya. We are convinced that further studies on archaeal DNA-topoisomerases will give us new gold nuggets that will contribute to have a better understanding of the DNA or RNA metabolism, with an additional interest when placed in an evolutionary perspective i.e., since LUCA, or even before!

## Author Contributions

All authors contributed to define the frame of this review and to write and draw the different parts.

## Conflict of Interest

The authors declare that the research was conducted in the absence of any commercial or financial relationships that could be construed as a potential conflict of interest.

## Footnotes

1 http://lifemap-ncbi.univ-lyon1.fr/

## Dedication

This article is dedicated to the memory of Maria Ciaramella who largely contributed to the understanding of the DNA-topoisomerases and the DNA repair field in Archaea.
